# The Structure of the Human Respiratory Syncytial Virus M2-1 Protein Bound to the Interaction Domain of the Phosphoprotein P Defines the Orientation of the Complex

**DOI:** 10.1128/mBio.01554-18

**Published:** 2018-11-13

**Authors:** Muniyandi Selvaraj, Kavestri Yegambaram, Eleanor J. A. A. Todd, Charles-Adrien Richard, Rachel L. Dods, Georgia M. Pangratiou, Chi H. Trinh, Sophie L. Moul, James C. Murphy, Jamel Mankouri, Jean-François Éléouët, John N. Barr, Thomas A. Edwards

**Affiliations:** a School of Molecular and Cellular Biology, University of Leeds, Leeds, United Kingdom; b Astbury Centre for Structural Molecular Biology, University of Leeds, Leeds, United Kingdom; c Unité de Virologie et Immunologie Moléculaires (UR892), INRA, Université Paris-Saclay, Jouy-en-Josas, France; Catholic University of America; Catholic University of America

**Keywords:** HRSV, M2-1, phosphoprotein

## Abstract

Human respiratory syncytial virus (HRSV) is a negative-stranded RNA virus that causes a globally prevalent respiratory infection, which can cause life-threatening illness, particularly in the young, elderly, and immunocompromised. HRSV multiplication depends on replication and transcription of the HRSV genes by the virus-encoded RNA-dependent RNA polymerase (RdRp). For replication, this complex comprises the phosphoprotein (P) and the large protein (L), whereas for transcription, the M2-1 protein is also required. M2-1 is recruited to the RdRp by interaction with P and also interacts with RNA at overlapping binding sites on the M2-1 surface, such that binding of these partners is mutually exclusive. The molecular basis for the transcriptional requirement of M2-1 is unclear, as is the consequence of competition between P and RNA for M2-1 binding, which is likely a critical step in the transcription mechanism. Here, we report the crystal structure at 2.4 Å of M2-1 bound to the P interaction domain, which comprises P residues 90 to 110. The P90–110 peptide is alpha helical, and its position on the surface of M2-1 defines the orientation of the three transcriptase components within the complex. The M2-1/P interface includes ionic, hydrophobic, and hydrogen bond interactions, and the critical contribution of these contacts to complex formation was assessed using a minigenome assay. The affinity of M2-1 for RNA and P ligands was quantified using fluorescence anisotropy, which showed high-affinity RNAs could outcompete P. This has important implications for the mechanism of transcription, particularly the events surrounding transcription termination and synthesis of poly(A) sequences.

## Abstract (precis)

Human respiratory syncytial virus (HRSV) is a leading cause of respiratory illness, particularly in the young, elderly, and immunocompromised, and has also been linked to the development of asthma. HRSV replication depends on P and L, whereas transcription also requires M2-1. M2-1 interacts with P and RNA at overlapping binding sites; while these interactions are necessary for transcriptional activity, the mechanism of M2-1 action is unclear. To better understand HRSV transcription, we solved the crystal structure of M2-1 in complex with the minimal P interaction domain, revealing molecular details of the M2-1/P interface and defining the orientation of M2-1 within the tripartite complex. The M2-1/P interaction is relatively weak, suggesting high-affinity RNAs may displace M2-1 from the complex, providing the basis for a new model describing the role of M2-1 in transcription. Recently, the small molecules quercetin and cyclopamine have been used to validate M2-1 as a drug target.

## INTRODUCTION

Human respiratory syncytial virus (HRSV) is the leading cause of serious respiratory tract infections in infants and poses a serious health threat to many other at-risk populations, such as the elderly and immunocompromised, causing an estimated 199,000 fatalities each year (1–3). There is currently no approved HRSV vaccine, and the only option to prevent HRSV-mediated disease is immunotherapy, which is expensive and incompletely protective.

HRSV is a pneumovirus classified within the Mononegavirales order and possesses a 15-kb genome comprising a single strand of negative-sense RNA, tightly wrapped with a virus-encoded nucleocapsid (N) protein to form a ribonucleoprotein (RNP) complex (4). The viral RNA (vRNA) genome contains 10 genes that are each flanked by transcription start and stop signals. These signals modulate the activity of the viral RNA-dependent RNA polymerase (RdRp) to generate a single 5′ capped and 3′ polyadenylated mRNA from each transcriptional unit. The RdRp must also replicate the vRNA genome, during which the transcription start and stop signals are ignored, resulting in the generation of a full-length complementary copy, known as the antigenome or cRNA (5).

The events that dictate whether the HRSV RdRp either transcribes or replicates are unclear, although the components of a replicating RdRp and a transcribing RdRp are different; the replicase requires the phosphoprotein (P), along with the large catalytic component (L), whereas the transcriptase additionally requires the M2-1 protein (6).

M2-1 is a multifunctional protein, which has been variously described as a transcription factor (7, 8), an antiterminator (9), a structural protein forming an RNP-associated layer within the virion (10, 11), and recently in a posttranscriptional function in which M2-1 associates with viral mRNAs (12), perhaps to influence translation. Of these, the best-characterized role is that of a transcription factor, in which M2-1 is thought to enhance polymerase processivity by suppressing transcription termination both intragenically (8, 13, 14) and intergenically (15, 16). In the case of intragenic transcription termination, M2-1 is thought to permit the generation of abundant full-length mRNAs rather than prematurely terminated products. In the case of intergenic antitermination, M2-1 is proposed to mask the gene end transcription termination signal and thus lead to the synthesis of abundant readthrough RNAs, which represent transcripts copied from two or more transcriptional units. By ignoring gene end termination signals, the RdRp may access more 3′ distal genes, as the transcription attenuation steps that occur at gene junctions will be bypassed. Recombinant HRSV in which the M2-1 gene has been deleted cannot be rescued, suggesting M2-1 is an essential protein for one or more of these activities (7, 8).

M2-1 is 194 amino acids in length (17), binds zinc atoms (9), and exists in solution as a tetramer (18). It is also dynamically phosphorylated at serine residues S58 and S61 to modulate its processivity function (19). The cellular phosphatase PP1 dephosphorylates these residues and specifically interacts with P at a critical RVxF motif (20), thus playing a critical role in cyclical interchange of the phosphorylated and dephosphorylated forms of M2-1 with P. M2-1 associates with the HRSV matrix protein (M) within assembled virions (21) and also infected cells (22), where they colocalize at sites of RNA synthesis inside punctate structures known as inclusion bodies (IBs) (23). In addition, M2-1 interacts with both viral RNA (19, 24) and P (25). This ability of M2-1 to interact with RNA and P is required for productive transcription, and each monomer within the tetramer can potentially interact with both an RNA molecule and P at an overlapping binding site (26, 27), suggesting that binding of these ligands at each binding site is mutually exclusive. M2-1 preferentially binds poly(A)-rich RNAs (26, 27), and its RNA binding activity correlates with the processivity function.

The overall fold of the M2-1 globular core (residues 58 to 177) was revealed by the nuclear magnetic resonance (NMR) solution structure (26), which also identified M2-1 residues involved in interactions with RNA and P through analysis of NMR spectral perturbations following ligand binding. The subsequently solved crystal structure of full-length M2-1 in its native tetrameric form (27) revealed atomic details of this globular core, as well as the oligomerization domain, the zinc-binding domain (ZBD), and also the reversible phosphorylation sites at S58 and S61.

The mechanism of M2-1-mediated processivity remains unclear. Current models postulate that that M2-1 interacts with nascent mRNAs cotranscriptionally, to stabilize the transcriptase complex, which ultimately leads to increased transcription of full-length mRNAs. In order to improve this model, a better structural characterization of the functional transcriptase complex is required. Here, we expressed and purified the M2-1 binding domain of HRSV P, subsequently allowing the formation of M2-1/P complex. We present the crystal structure of M2-1 in complex with the P interaction domain, at a resolution of 2.4 Å, which defines the orientation of the M2-1 and P moieties in the heteromultimeric complex. Functional dissection of residues that comprise the M2-1/P binding site using minigenome analysis confirmed the critical nature of the M2-1/P interaction. Lastly, after analysis of the relative binding affinities of the M2-1 ligands, we present an updated model of the role of M2-1 in transcription.

## RESULTS

### Delineation of the HRSV M2-1 interaction domain within P.

NMR analysis and secondary structure prediction indicate that HRSV P comprises largely unstructured N- and C-terminal sequences flanking short ordered regions involved in homo- and heterotypic interactions, including an oligomerization domain (residues 126 to 163), M2-1, and N and L interaction sites, as well as a distinct site that interacts with N in assembled RNPs (28–37). Previous work by others using minigenome analysis has identified P residues important for M2-1 interactions, comprising residues 100 to 120 (25). More recently residues 90 to 112 were implicated in M2-1 binding using NMR, whereas residues 93 to 110 were defined as being sufficient for M2-1 binding using a glutathione *S*-transferase (GST)-pulldown assay (20). Here we sought to confirm independently the identity of P residues involved in the M2-1 interaction, prior to embarking on further biochemical and structural studies.

**FIG 1:**
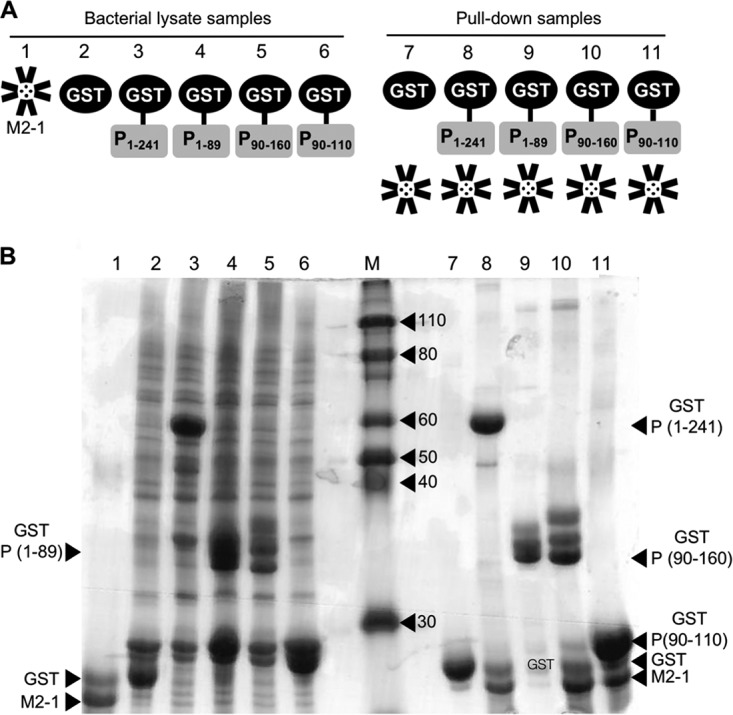
Expression of GST-tagged P proteins, and their interactions with HRSV M2-1. (A) Schematic of GST fusion constructs, with P moieties shown as yellow rectangles, GST as a black circle and M2-1 as a cartoon representation of the tetramer atomic model. (B) SDS-PAGE analysis of lysates and pulldowns, with lanes labeled to correspond with the schematic above. Proteins were visualized by Coomassie staining. Lanes 1 to 6 contain protein expression lysates. Lanes 7 to 11 show analysis of GST pulldowns, in which GST-P constructs were incubated with full-length M2-1, and eluted bound proteins were analyzed and visualized by SDS-PAGE, followed by Coomassie staining.

Various full-length and truncated P constructs (Fig. 1A) were expressed as GST fusion proteins in bacterial cells, bound to glutathione resin, and incubated with full-length native tetrameric M2-1. The resulting complexes were analyzed by SDS-PAGE (Fig. 1B). Efficient M2-1 binding and subsequent pulldown were retained for constructs P1–241, P90–160, and P90–110, whereas no binding was evident for N-terminal construct 1–89. These results are in close agreement with previous findings, and show that P residues 90 to 110 are sufficient to bind M2-1.

### Crystal structure of HRSV M2-1 bound to P90–110.

Following biochemical characterization of M2-1/P binding, we next sought to understand the molecular basis for this interaction by obtaining high-resolution structural data of the M2-1/P complex using X-ray crystallography. Native M2-1 was purified as tetramers and incubated with P90–110 in a 1:1 molar ratio prior to concentration and crystallization trials. The resulting crystals exhibited a needle morphology, in contrast to the plate-like crystals previously described for M2-1 alone, and X-ray diffraction data were collected with a maximum resolution of 2.4 Å (Table 1). The data set was processed and the structure of the complex solved by molecular replacement with one monomer of M2-1 (PDB no. 4C3B) as the reference search model. The electron density difference maps revealed a continuous area of density not accounted for by M2-1 and which appeared to be a peptide that exhibited helical secondary structure. The peptide density was confirmed by calculating an omit map. The P90–110 peptide was manually built into this density, with side chains modeled after several rounds of refinement.

**TABLE 1:** Crystallographic data

Parameter^*a*^	Result for parameter shown^*b*^
Wavelength (Å)	0.98
Space group	P2_1_2_1_2
Unit cell dimensions *a*, *b*, *c* (Å)	96.55, 116.52, 72.63
α = β = γ (°)	90
No. of total reflections	402,467 (29,942)
No. of unique reflections	32,012 (2,309)
Resolution shells (Å)	
Low	74.35–2.42
High	2.48–2.42
I/σ⟨*I*⟩	9.5 (2.2)
*R*_merge_ (%)	15 (95)
*R*_pim_ (%)	6.4 (39)
*R*meas (%)	16.6 (0.96)
Solvent content (%)	47
*V_*m*_* (Å^3^/Da)	2.32
No. of molecules/AU	4 monomers
Completeness (%)	100 (98.6)
Multiplicity	12.6
*R*_work_ (%)	22
*R*_free_ (%)	28
No. of atoms used in refinement	6,022
No. of water molecules	229
Mean *B* values (Å^2^)	57
RMSD	
Bond lengths (Å)	0.1
Bond angle (°)	1.7
Ramachandran plot (%)	
Preferred region	95.81
Allowed region	3.47
Outlier	0.72
MolProbity score	
Clashscore for all atoms	7.5
Score for protein geometry	2.6

a Shown are HRSV M2-1/P90–110 complex data represented by PDB code 6G0Y. *R*_pim_, precision-indicating *R*_merge_; *V_m_*, specific volume (Matthews coefficient); AU, asymmetric unit.

b Values in parentheses represent the highest-resolution shell.

**FIG 2:**
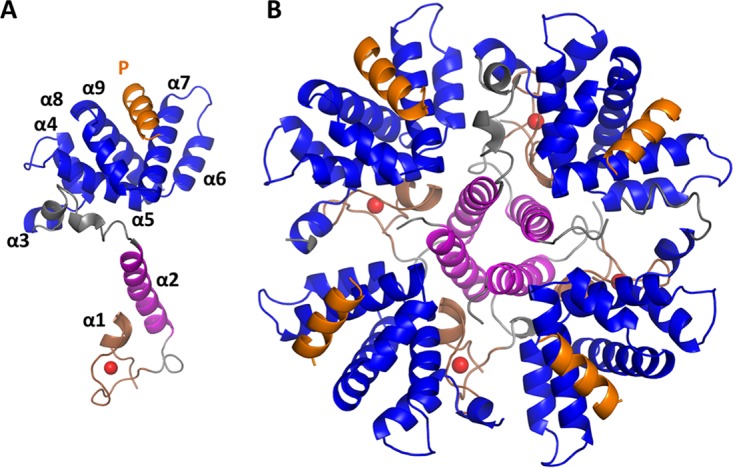
Crystal structure of the M2-1/P complex. (A) The M2-1 monomer in the M2-1/P complex is shown with alpha helices numbered sequentially from N- to C termini. The N-terminal zinc-binding domain is shown with a coordinated zinc ion (red sphere), the oligomerization helix is shown in pink, the core domain is shown in blue, and the P90–110 peptide is shown in orange. (B) The M2-1/P complex with M2-1 in its tetramer state, color coded as in panel A. Models were constructed using PyMol.

The cocrystal structure of M2-1/P90–110 was solved in the P2_1_ 2_1_ 2 space group, with a single tetramer in the asymmetric unit comprising four copies of M2-1, each bound to a single molecule of P90–110 with 1:1 stoichiometry (Fig. 2A). Residues 97 to 109 of the P90–110 peptide were reliably discernible in the density difference maps, and all four P90–110 molecules were orientated with equivalent pose on the surface of the respective M2-1 monomers. The orientation of the four P90–110 molecules on the M2-1 tetramer defines the relative orientation of M2-1 and P in the heterotypic complex. On the basis of this model, we propose that, in the absence of RNA, each M2-1 tetramer associates with a single P tetramer, with all four P binding sites of M2-1 being occupied.

**FIG 3:**
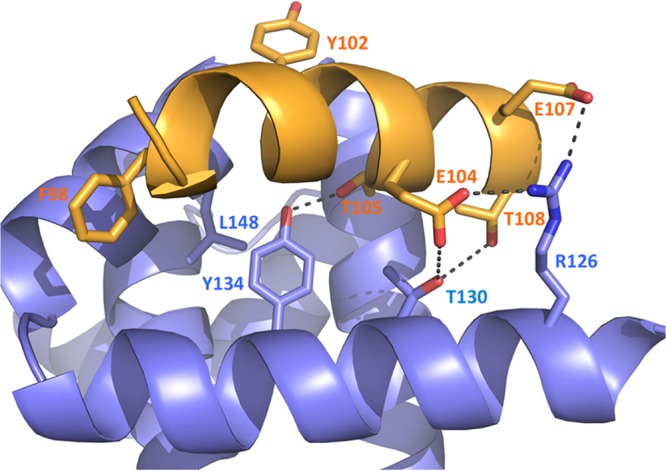
Details of M2-1/P electrostatic interactions as revealed by the M2-1/P90–110 cocrystal structure. M2-1 residues are labeled in blue, and P peptide residues are labeled in orange. Hydrophobic residues mutated in replicon experiments are also highlighted. The figure was generated using PyMol (PyMOL Molecular Graphics System, version 1.7.2.3; Schrödinger, LLC.).

The P90–110 peptide forms a single alpha helix that lies along a cleft formed by three alpha helices (α7, α8, and α9) of M2-1 that build up one face of its globular core (Fig. 2B). The P90–110 peptide binds to this face in a position that includes residues previously identified as important for binding P and also binding RNA. Here, we reveal the atomic details of interactions that drive these associations. The M2-1/P interface involves ionic, hydrophobic, and hydrogen bond interactions (Fig. 3), and of the 16 residues of M2-1 that lie within 4 Å and interact with P90–110, 15 of these are conserved across the various respiratory syncytial orthopneumoviruses within the Pneumoviridae family (see Fig. S1 and S2 in the supplemental material). P90–110 is orientated such that hydrophilic side chains of K100, K103, E104, and E107 are facing mostly toward the solvent, whereas hydrophobic residues P97, F98, L101, and I106 are mostly facing toward the cleft within the M2-1 core (see Fig. S3 in the supplemental material).

10.1128/mBio.01554-18.1 FIG S1 Alignment of the amino acid sequences of multiple pneumovirus P proteins. The shaded region is from positions 90 to 110, with M2-1-interacting residues marked with arrows. Alignment was generated by Clustal Omega. UniProt accession numbers: human RSV, P04545; bovine RSV, P29792; ovine RSV, Q84132; murine pneumonia virus, Q5MKM1; human metapneumovirus, Q6WB97; avian metapneumovirus, Q2Y2M2. Download FIG S1, TIF file, 1.6 MB. Copyright © 2018 Selvaraj et al. 2018 Selvaraj et al. This content is distributed under the terms of the Creative Commons Attribution 4.0 International license.

10.1128/mBio.01554-18.2 FIG S2 Alignment of the amino acid sequences of multiple pneumovirus M2-1 proteins using Clustal Omega. UniProt accession numbers: human RSV, P04545; bovine RSV, P29792; ovine RSV, Q84132; murine pneumonia virus, Q5MKM1; human metapneumovirus, Q6WB97; avian metapneumovirus, Q2Y2M2. Residues of M2-1 that are positioned between 2.5 to 4 Å from the P-peptide are marked with an arrow. Download FIG S2, TIF file, 1.6 MB. Copyright © 2018 Selvaraj et al. 2018 Selvaraj et al. This content is distributed under the terms of the Creative Commons Attribution 4.0 International license.

10.1128/mBio.01554-18.3 FIG S3 Omit map density for P peptide (green mesh) allows unambiguous positioning of the peptide. Download FIG S3, TIF file, 2.4 MB. Copyright © 2018 Selvaraj et al. 2018 Selvaraj et al. This content is distributed under the terms of the Creative Commons Attribution 4.0 International license.

R126 of M2-1 interacts with P90–110 residues E104, E107, and T108 by a combination of ionic and hydrogen bond interactions. M2-1 residue T130 interacts with T108 of P90–110, whereas Y134 interacts with T105. There is a total of 10 plausible hydrogen bonds, with R126 making what appear to be important salt bridges to E104 and E107 (Fig. 3). There are a multitude of hydrophobic interactions along the length of the P peptide, highlighted by L148 of M2-1 packing against L101 and Y102 of the P peptide.

There are no major changes to the backbone positions of M2-1 upon binding of the P peptide when comparing the P bound complex (PDB no. 6G0Y) with M2-1 alone (PDB no. 4C3B). The root mean square deviation (RMSD) on superposition of 158 carbon alphas is only 1.097 Å, suggesting very little structural rearrangement on P binding. The M2-1 side chains of R126 and Y134 move to satisfy the hydrogen bonds, as described in Fig. 3, but other than that, there is little movement of side chains required to accommodate the P peptide. The loop on M2-1 containing S58 and S61 (19), which are dynamically phosphorylated in HRSV-infected cells, appears to be slightly more ordered (the electron density for this region is clearer) in the complex crystal structure. However, whether this is because the loop is ordered upon P binding or otherwise results from crystal packing is currently unknown.

### Testing the importance of residues within the M2-1/P interface for mRNA transcription.

The crystal structure of the M2-1/P90–110 complex revealed molecular details of the M2-1 and P interface, defining the contacts that drive this interaction. We next tested the relevance of these contacts using an HRSV minigenome system that provides a quantitative measure of the functionality of the HRSV polymerase complex. In this system, mammalian cells are transfected with cDNAs expressing the protein components of the HRSV polymerase complex, namely N, P, L, and M2-1, along with a cDNA expressing a minigenome. The minigenome is bicistronic, with two transcription units separated by an authentic HRSV gene junction, with the downstream gene encoding green fluorescent protein (GFP) as a reporter protein. Expression of enhanced GFP (eGFP) in this system is entirely dependent on functionality of the polymerase complex, including the M2-1/P interaction, and is detected and quantified by real-time analysis of eGFP fluorescence in living cells.

**FIG 4:**
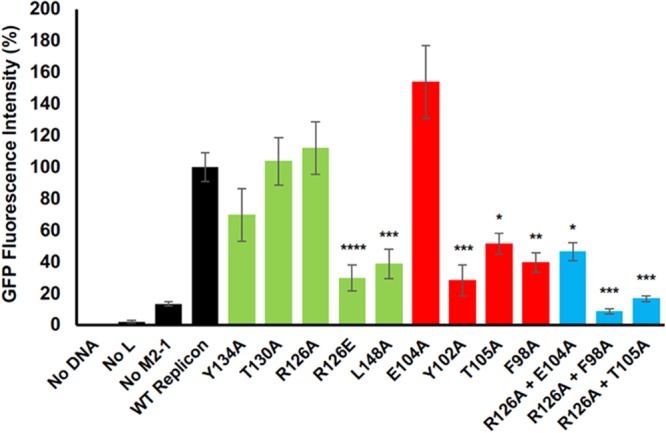
Examination of the role of M2-1 and P residues in forming a functional M2-1/P complex active for HRSV gene expression. Critical residues that comprise the M2-1/P interface were altered, and the corresponding M2-1 and P proteins were used to reconstitute the HRSV transcriptase complex, along with unaltered N and L proteins, using a minigenome system. The ability of M2-1 mutants (green bars), P mutants (red bars), or double M2-1/P mutants (blue bars) to form a functional complex able to support transcription of a GFP reporter gene from the supplied minigenome was quantified by counting the intensity of GFP expression in minigenome-harboring cells. The histogram shows relative GFP intensity, normalized to GFP expression from cells transfected with wild-type minigenome components. Significance values: ****, *P* ≤ 0.0001; ***, *P* ≤ 0.001; **, *P* ≤ 0.01; * *P* ≤ 0.05.

On the basis of the crystal structure of the M2-1/P90–110 complex, the cDNAs expressing M2-1 and P proteins were altered in order to mutate selected amino acids at the interaction interface. M2-1 mutants comprised R126A, R126E, T130A, Y134A, and L148A, whereas the P mutants were F98A, Y102A, E104A, and T105A. These altered cDNAs were substituted for the corresponding wild-type (WT) cDNAs in minigenome experiments, and in addition, selected pairings of cDNAs expressing mutant M2-1 and P proteins were also transfected to examine the combined effect of simultaneously altering interacting residues from both components of the M2-1/P complex (Fig. 4).

Of the M2-1 mutants, only R126E and L148A resulted in significant disruption of minigenome activity, with eGFP fluorescence values reduced to approximately 30% that of WT M2-1. The reduction in activity of the R126E mutant is consistent with the role of R126 in forming electrostatic interactions with multiple P residues, including E104 and E107 (Fig. 3). In particular, disruption of the interaction between R126 and E107 likely contributes in major part to loss of minigenome activity due to the close proximity of these interacting groups (2.59 Å). The significant reduction in minigenome activity exhibited by the L148A substitution is consistent with the observed hydrophobic interaction of this residue with L101 from P90–110.

Of the P mutants, alanine substitutions at residues F98, Y102, and T105 resulted in significantly reduced minigenome activity, consistent with these residues playing important roles in mediating the M2-1/P interaction. Interestingly, the E104A substitution had no significant effect on polymerase function. We suggest here that alternative residues located on the M2-1 surface can still make other interactions to keep P in place. The simultaneous incorporation of altered M2-1 and P proteins had an additive effect on minigenome activity (Fig. 4), with most reduced activity exhibited by combining mutants R126E and F98A, which reduced eGFP expression to that of the minus M2-1 control. Taken together, the results from the minigenome analysis validate the structural analysis of the M2-1/P interface, confirming that multiple interacting residues, as described above, are important for maintaining the integrity of the complex.

### Quantification of the M2-1/P90–110 interaction using fluorescence anisotropy.

The results of the previous section showed binding of P90–110 involves residues of M2-1 previously identified as interacting with RNA (26, 27), further establishing that RNA and P occupy overlapping binding sites on the M2-1 surface. Therefore, we next wanted to quantify and compare the relative affinities of M2-1 for both P90–110 and RNA, to establish whether one ligand may outcompete the other, which would have functional consequences during the HRSV life cycle.

Previously, we determined that native tetrameric M2-1 exhibited various affinities for RNAs, depending on both their size and sequence (27); positive-sense gene end sequences (A-rich) exhibited affinities with dissociation constant (*K_d_*) values ranging between 46.5 and 263 nM, whereas negative-sense gene end sequences (U-rich) exhibited significantly reduced affinities, with *K_d_* values between 860 and >10,000 nM. The highest recorded affinity was for poly(A) RNA oligomers 13 nucleotides in length (A13; *K_d_* =19 nM) (27).

**FIG 5:**
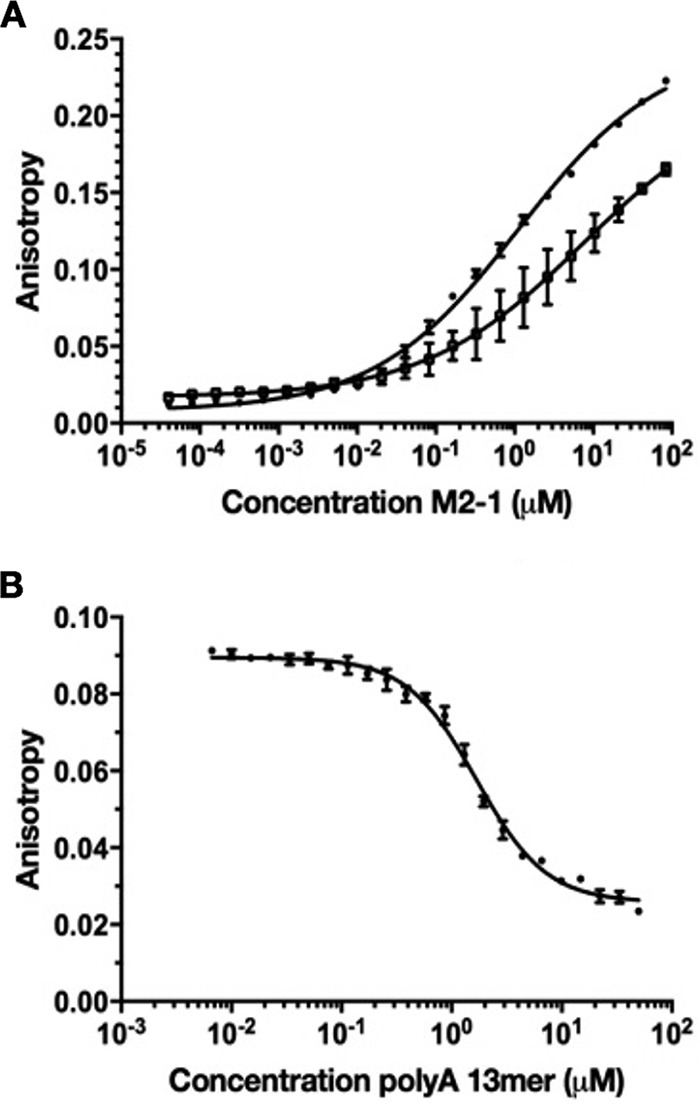
Fluorescence anisotropy measurements of M2-1/P interactions. (A) Direct binding of M2-1 with fluorescein-labeled P90–110. Data points are from experiments performed in triplicate, with a P90–110 concentration of 10 nM. (B) FA competition assay in which fluorescein-labeled P90–110 was outcompeted for M2-1 binding by unlabeled poly(A) RNA 13-mer.

The affinity of P90–110 binding to M2-1 was examined by fluorescence anisotropy (FA) using tetrameric M2-1 and a fluorescein-labeled P90–110 peptide (Fig. 5A). This peptide bound weakly to M2-1, with an apparent *K_d_* of 7.5 µM, which was nearly 3 orders of magnitude lower than that previously determined for A13 and lower than those for almost all other RNAs previously tested (27). On the basis of this finding, we next examined whether various RNA sequences were able to outcompete M2-1/P binding. This was achieved by measuring FA of M2-1 bound to fluorescein-labeled P90–110 in the presence of increasing concentrations of unlabeled RNA ligand, which revealed P90–110 was outcompeted by A13 with a 50% inhibitory concentration (IC_50_) of 1.7 µM (Fig. 5B). In contrast, none of the RNAs tested could be outcompeted by P90–110 (data not shown). These findings suggest for each binding site, the binding of P or RNA is mutually exclusive and that binding of an RNA sequence that exhibits high affinity, such as an A-rich gene end or poly(A) tail, would likely displace a monomer of P from the M2-1 surface.

## DISCUSSION

**FIG 6:**
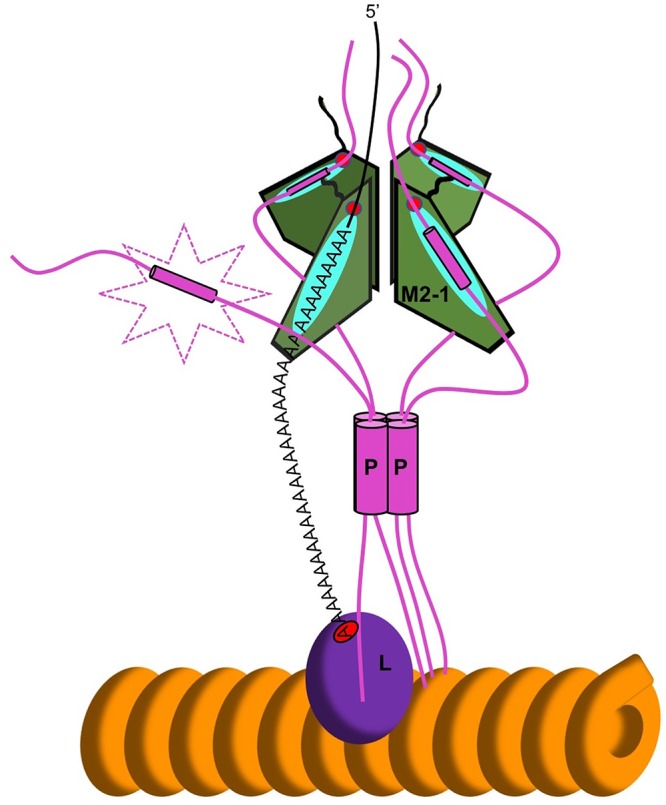
Schematic representation of the interaction between M2-1 and P in the context of the transcribing HRSV polymerase complex. The orange helix represents RNP. L (red) and P (blue) form the complex that generates mRNA. M2-1 (gray) is recruited to the complex via P. The P90–110 peptide is displaced from one of the P binding sites on M2-1 by A-rich RNA.

The elucidation of the relative orientation of the M2-1 and P monomers now allows us to add further detail to the model that describes the architecture of the HRSV RdRp transcriptase complex and the molecular basis for M2-1 function. Our data show that P90–110 orientates within a cleft on the surface of M2-1 such that its N terminus faces the N-terminal surface of M2-1, adjacent to the zinc-binding domain (ZBD). The M2-1/P90–110 cocrystal structure identifies amino acid side chains within the M2-1/P interface that drive the formation of this complex. This orientation of P was previously proposed, based on mutagenesis and NMR interaction data (20), and here this orientation is explicitly defined. If the various interacting domains of P are arranged in a linear fashion, this would also dictate that the N terminus of M2-1 would face away from both the RdRp and the RNP template, which is consistent with the RNP binding site at the P C terminus (Fig. 6). Consequently, as the RNA and P binding sites overlap, this would also define the orientation of the RNA binding surface relative to the polymerase active site and RNA exit channel. This coincides with the orientation of the short deoxyoligonucleotide that was cocrystallized with M2-1 from HMPV, for which the 5′ end was in contact with the N-terminal face of M2-1 alongside the ZBD, with the RNA 3′ end in contact with the opposing C-terminal face (38). Our model proposes that the RNA binding surface of M2-1 is positioned such that it would be able to interact with a nascent RNA as it emerged from the RdRp (Fig. 6).

The higher affinity of M2-1 for almost all tested RNAs over P90–110 (as much as 1,000-fold higher) suggests that nascent RNAs will likely displace P from the shared binding site, provided the RNA sequence is A-rich. As each RdRp only transcribes one RNA molecule at a time, it is likely that only one P monomer will be displaced from the RNA/P binding site, allowing M2-1 to associate with both P and RNA simultaneously in a 4:3:1 stoichiometry, as suggested in Fig. 6. However, we cannot formally rule out the possibility that the nascent RNA adopts a folded state that can either interact with more than one RNA binding site on M2-1 or otherwise disrupt P binding by steric hindrance.

Determining the fate of this tripartite M2-1/P/mRNA complex will be critical in understanding the mechanism of action of M2-1. The ability of M2-1 to bind most tightly to A-rich sequences, such as those found at the 3′ end of the mRNA [e.g., at the gene end or the poly(A) tail itself] leads us to speculate that M2-1 acts following the emergence of these high-affinity sequences from the RdRp active site, which is necessarily during transcription termination. Therefore, one possibility is that the nascent and newly terminated mRNA could bind to M2-1 to form the tripartite M2-1/P/RNA complex, and on account of the high affinity of M2-1 for these A-rich 3′ sequences, the released mRNA could displace M2-1 from the P/L RdRp complex. The likelihood of this displacement will depend on the relative affinities of M2-1 for the single strand of nascent RNA in relation to three remaining molecules of P. In view of this, it is important to acknowledge that our FA competition assay measures the assembly and disassembly of the M2-1/P complex in which M2-1 is in its native tetramer form, whereas P90–110 exists as a monomer rather than the native tetramer form of P. In the context of a tetramer/tetramer M2-1/P interaction, it is likely that M2-1/P affinities are influenced by avidity and that the overall affinity of M2-1 for P tetramers will be significantly higher than for the monomeric P peptide, a topic that has recently been explored by others (39).

An important clue to the fate of terminated HRSV mRNAs was recently revealed, in which M2-1 was identified in association with polyadenylated mRNAs within specialized compartments termed inclusion body-associated granules (IBAGs) (12). IBAGs are associated with inclusion bodies that are the putative sites of HRSV RNA synthesis. Here, M2-1 is proposed to play a posttranscriptional role in the expression of the HRSV mRNAs, possibly in stabilizing (or protecting) mRNAs, helping their export to the cytosol, and/or enhancing their translation. Importantly, within the IBAGs, M2-1/mRNA complexes are not associated with P, and this is consistent with the above scenario, where following transcription termination, the released mRNA displaces M2-1 from P. This would also exclude the ribonucleoprotein from the IBAG, as is observed.

While this possibility is attractive and explains the colocalization of M2-1 with mRNAs, one particular aspect of this model presents a major incongruity that we cannot currently reconcile: this relates to M2-1 abundance. Critically, for each mRNA to dissociate from the RdRp along with a bound M2-1 tetramer, the abundance of M2-1 would need to exceed that of all mRNAs combined by 4-fold. According to the long established dogma that describes the Mononegavirales transcription gradient (40), M2-1 gene expression is expected to be relatively low, and examination of HRSV transcript abundance by next-generation sequencing would appear to back this up (41). One possible explanation for this apparent discord is that M2-1 is rather stable and may also be recycled by the dynamic phosphorylation, allowing M2-1 in IBAGs to return to the site of transcription to associate with further mRNAs.

A deficit in M2-1 abundance would be particularly evident during the process of primary transcription, if the RdRp bound to the infecting vRNA is the only source of M2-1 in the newly infected cell. Once the first (NS1) mRNA was transcribed, dissociation of M2-1 from the resident RdRp would prevent further productive transcription, as no more M2-1 would be available as a replacement. However, if M2-1 is also associated with the matrix in the virion, as proposed (10), then the virion may bring sufficient M2-1 to an infected cell to start effective transcription.

An alternative outcome of M2-1/RNA binding is that the terminated mRNA may be released in an unbound form, such that the association of the M2-1/P complex alongside the L protein remains intact. In this scenario, it is interesting to speculate why M2-1 might associate with 3′ mRNA or poly(A) sequences and how this may relate to the functions of M2-1 in processivity, antitermination, or translation. One possibility is that M2-1 plays a stimulatory role in mRNA polyadenylation. Perhaps the 3′ end of a nascent transcript interacts with M2-1, which in turn induces the P/L RdRp complex to switch from a processive templated polymerization mode into a reiterative polymerization mode, in which the short gene end U-tract is repeatedly copied to generate the 3′ poly(A) tail.

Such a role would be consistent with previous reports of truncated mRNAs detected in the absence of M2-1, as tail-less RNAs would be rapidly degraded. In addition, stimulation of polyadenylation would also be consistent with the translation role of M2-1 within IBAGs, with the poly(A) tail facilitating translation through mRNA circularization. Further structural, biochemical, and cellular investigations of M2-1 and its binding partners will aid in resolving these critical questions surrounding the role of M2-1 in HRSV gene expression.

## MATERIALS AND METHODS

### GST-tagged HRSV P expression and purification.

A cDNA of the P gene (A2 strain) was cloned into the pGEX-6P-2 plasmid to allow expression in Escherichia coli BL21(DE3) of full-length and truncated P sequences fused at their N terminus to the GST affinity tag. PCR was used to engineer all truncations within the P open reading frame (ORF), leaving the GST tag unaffected. Bacteria were transformed with pGEX-6P-2-derived plasmids, grown to an optical density at 600 nm (OD_600_) of 0.8, at which time expression was induced by the addition 0.1 mM IPTG (isopropyl-β-d-thiogalactopyranoside) followed by further incubation at 37°C for 18 h. Cells were pelleted by centrifugation and stored at −80°C.

### GST pulldown assay.

GST protein and GST-P fusion proteins were expressed as described above. Cell pellets from 10-ml bacterial cultures were suspended in 1 ml pulldown buffer (25 mM Tris/HCl [pH 7.4], 150 mM NaCl, 1 mM dithiothreitol [DTT]) and sonicated for 2 min on ice. Lysates were clarified by centrifugation.

Glutathione Sepharose 4B (GE Healthcare) was prepared by adding 200 μl to microcentrifuge tubes. The resin was washed with water and equilibrated with pulldown buffer using the batch method. Clarified lysates of GST or GST-P were added to the resin and incubated at room temperature for 5 min. The lysates were then removed, and the resin was washed three times with pulldown buffer. Purified M2-1 protein (1 mg) was added to the resin-bound GST-P proteins and incubated at room temperature for 5 min. The resin was washed extensively, and the resin slurry was mixed with SDS loading dye, boiled, and analyzed by SDS-PAGE.

### HRSV M2-1 protein expression and purification.

A cDNA representing the M2-1 ORF (strain A2) was inserted into plasmid pGEX-6P-2 to allow expression of M2-1 fused at its N terminus to GST. M2-1 was separated by the PreScission protease cleavage sequence to allow removal of the GST moiety, as described previously (27). Starter cultures of transformed E. coli BL21(DE3) Gold cells were grown at 37°C until an OD_600_ of 0.8 was reached, after which expression was induced by the addition of 0.5 mM IPTG, and cultures were supplemented with 50 nM ZnSO_4_. Cells were maintained at 18°C for 16 h and pelleted by centrifugation. The cells were then resuspended in lysis buffer (25 mM Tris-HCl [pH 7.4], 1 M NaCl, 1 mM DTT, 5% glycerol, 0.1% Triton X-100, protease inhibitor tablet [Roche]) and subjected to 4 cycles of freezing/thawing followed by the addition of 1 μg/ml RNase/DNase. Lysates were clarified by centrifugation, and the supernatant was applied to glutathione Sepharose 4B resin by gravity flow. Following extensive washes using lysis buffer and cleavage buffer (25 mM Tris-HCl [pH 7.4], 150 mM NaCl, 1 mM DTT, 5% glycerol), the fusion protein was cleaved on the column using PreScission protease, followed by elution with 2 column volumes (CVs) of cleavage buffer. Eluted protein was subjected to ion-exchange chromatography using SP Sepharose resin in low-salt buffer (25 mM Tris-Cl [pH 7.4], 50 mM NaCl, 5% glycerol, 1 mM DTT). Following washes with 5 CVs of low-salt buffer, proteins were eluted in a mixture of 25 mM Tris-Cl (pH 7.4), 600 mM NaCl, 5% glycerol, and 1 mM DTT. Fractions containing purified M2-1 protein were concentrated using a 10-kDa Vivaspin concentrator (GE Healthcare) and flash frozen in liquid nitrogen.

### Crystallization of the M2-1/P90–110 complex.

M2-1 was further purified for crystallization experiments by size exclusion chromatography. The protein sample was applied to a Superdex 75 column equilibrated in a mixture of 25 mM Tris-HCl (pH 7.4), 150 mM NaCl, and 1 mM DTT. Collected fractions were concentrated using 10-kDa molecular weight cutoff filters to be used in downstream experiments.

Residues 90 to 110 of the HRSV P (strain A2) were synthesized by ProteoGenix in an unlabeled form, and resuspended in a mixture of 25 mM Tris-HCl (pH 7.4) and 150 mM NaCl. Purified M2-1 was mixed with P90–110 in a 1:1 molar ratio and subsequently concentrated to 8 mg/ml. Crystallization trials were performed using a Formulatrix NT8 liquid handling robot to set up sparse matrix screens in an MRC 96-well plate robot using the sitting drop vapor diffusion method at 18°C. Crystallization conditions were screened for crystal formation using the Formulatrix Rockimager 1000. Crystals exhibiting both the previously reported plate morphology as well as an alternative needle morphology were obtained. Optimization was focused on the needle-like crystal conditions, which were optimized for polyethylene glycol (PEG) concentration to 0.2 M trimethylamine *N*-oxide dihydrate, 0.1 M Tris-HCl (pH 8.5), and 2% PEG MME 2000. Crystals were picked with appropriately sized nylon loops (Hampton Research) and cryo-cooled in mother liquor substituted with 5% glycerol, 5% PEG 400, 5% 2-MPD (2-methyl-2,4-pentandediol), and 5% ethylene glycol.

### Data collection and structure solution.

Data for all M2-1/P crystals was collected at the Diamond Light Source, beamline I02, to a maximum resolution of 2.4 Å, and all X-ray data were integrated into space group P2_1_2_1_2. All crystallographic calculations and refinement were performed using CCP4 suite (42), and the structure was solved by molecular replacement.

### Mutagenesis and minigenome assay.

The ability of the HRSV M2-1 and P proteins to functionally interact was measured using the previously described HRSV minigenome assay (26). Briefly, BSRT7 cells in 6-well plates were transfected using Lipofectamine 2000 (Thermo Fisher) in Opti-MEM (Thermo Fisher) with plasmids expressing HRSV N, P, L and M2-1 proteins, along with a plasmid expressing an RNA minigenome. This RNA template possessed intact HRSV promoter regions and the M/SH gene junction separating two transcriptional units. Expression of eGFP from the downstream gene was dependent on expression of M2-1 and P, as well as their ability to interact, and was measured in live cells after 24 h using an Incucyte Zoom (Essen Bioscience). Levels of eGFP expression were used to quantify the total number of eGFP-expressing cells in each well, which was normalized to complete transfections with unaltered HRSV plasmids.

### RNA binding and competition studies.

The ability of P90–110 and various RNA sequences to bind to M2-1 either individually or in competition was examined using fluorescence anisotropy (FA). Oligoribonucleotides of various sequences were synthesized with a 3′ fluorescein label, all in the 2′ ACE protected form (Dharmacon) whereas peptide P90–110 was synthesized by ProteoGenix with a fluorescein labeled at its N terminus. FA assays were carried out in 384-well format, in RNA binding buffer (20 mM Tris-Cl [pH 7.5], 150 mM NaCl, 0.01% Triton X-100). Direct binding of either P90–110 or RNA oligomers to M2-1 was assessed using 10 nM fluorescein-labeled ligand and increasing concentrations of M2-1 protein (0.1 nM to 300 μM). Following a 30-min incubation at room temperature, polarization was measured using a EnVision 2,103 multilabel plate reader (Perkin Elmer) equipped with a 480-nm excitation filter and 530-nm S- and P-channel emission filters. Experiments were performed in triplicate, and data were expressed as the fraction of RNA bound, plotted against protein concentration and fitted by standard logistic regression using OriginPro 8.6 (Origin Lab), as previously described. Dissociation constants (*K_d_*s) were averaged from the *K_d_* calculated from each triplicate data set. Competition studies were performed by incubating 1 μM M2-1 and 10 nM fluorescein-labeled P90–110 at room temperature for 10 min, after which serial dilutions of unlabeled RNA competitor was added, and the plate was incubated for a further 30 min at room temperature, prior to analysis as described above.

### Accession number(s).

The X-ray structure of the M2-1:P protein complex has been submitted to the PDB database under accession no. 6G0Y.

10.1128/mBio.01554-18.4 FIG S4 M2-1/P hydrophobic interactions. M2-1 residues are labeled in blue, and P peptide residues are labeled in orange. The figure was generated using LigPlot+ 2.0 (R. A. Laskowski, M. B. Swindells, J Chem Inf Model 51:2778–2786, 2011, https://doi.org/10.1021/ci200227u). Download FIG S4, TIF file, 0.1 MB. Copyright © 2018 Selvaraj et al. 2018 Selvaraj et al. This content is distributed under the terms of the Creative Commons Attribution 4.0 International license.

10.1128/mBio.01554-18.5 FIG S5 Orientation of side chains of residues within P90–110. Shown is a surface representation of the M2-1 tetramer in gray, with one monomer colored in cyan, on which the RNA binding surface is colored in yellow and the RNA/P binding surface is colored in coral. The P90–110 peptide bound to M2-1 is shown as an alpha helix with hydrophilic charged residues colored in red facing outwards towards the solvent, with side chains of K100, K103, E104, and E107 shown. Side chains of hydrophobic residues P97, F98, L101, and I106 face the M2-1 molecule and are shown in green. Download FIG S5, TIF file, 1.1 MB. Copyright © 2018 Selvaraj et al. 2018 Selvaraj et al. This content is distributed under the terms of the Creative Commons Attribution 4.0 International license.
