# Supplementary figures and images for: The Structure of the Human Respiratory Syncytial Virus M2-1 Protein Bound to the Interaction Domain of the Phosphoprotein P Defines the Orientation of the Complex
Source: mBio. 2018 Nov 13;9(6):e01554-18. doi: 10.1128/mBio.01554-18 (PMC6234862; doi:10.1128/mBio.01554-18)

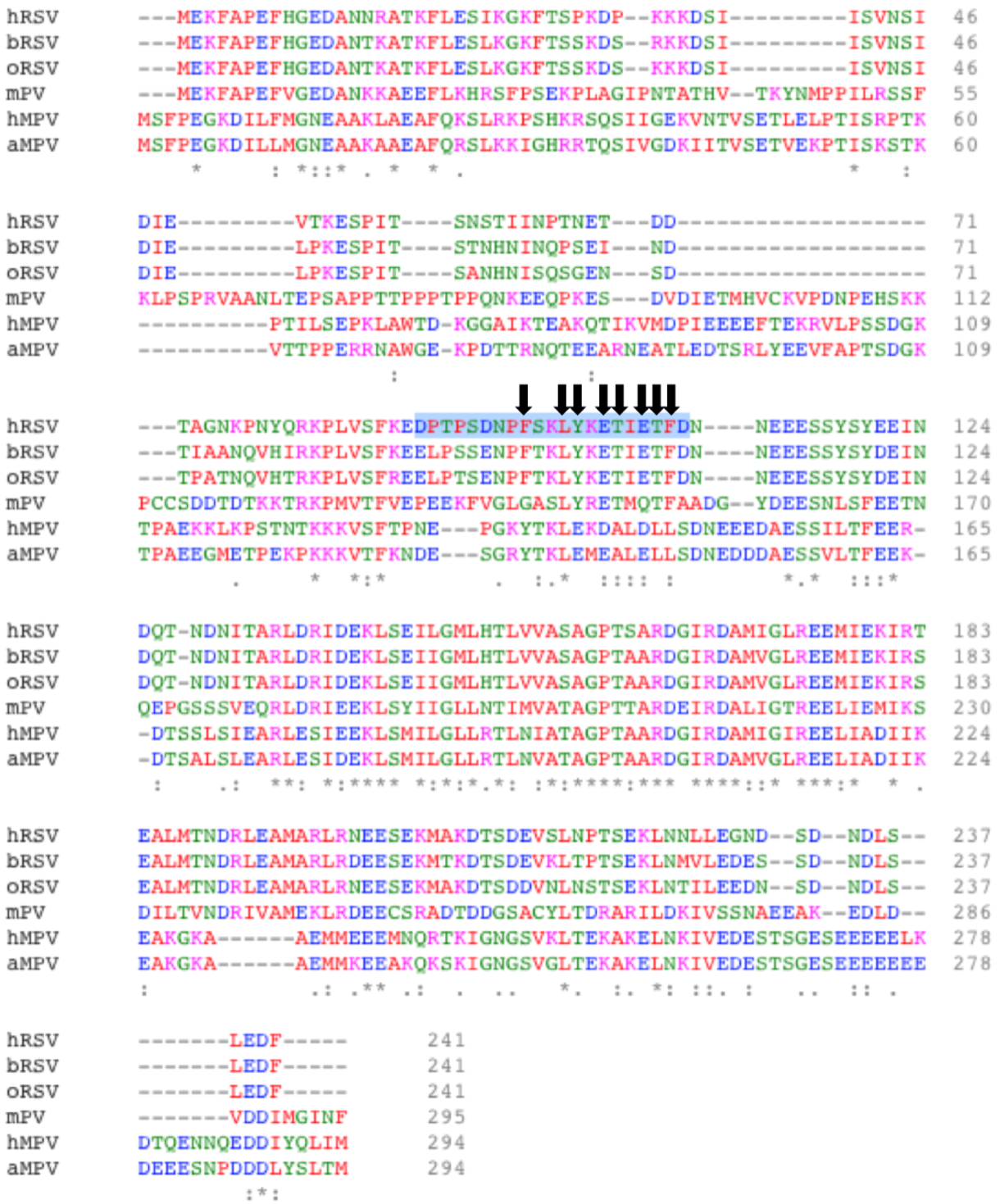

Supplement: FIG S1 [file mbo005184162sf1.tif]

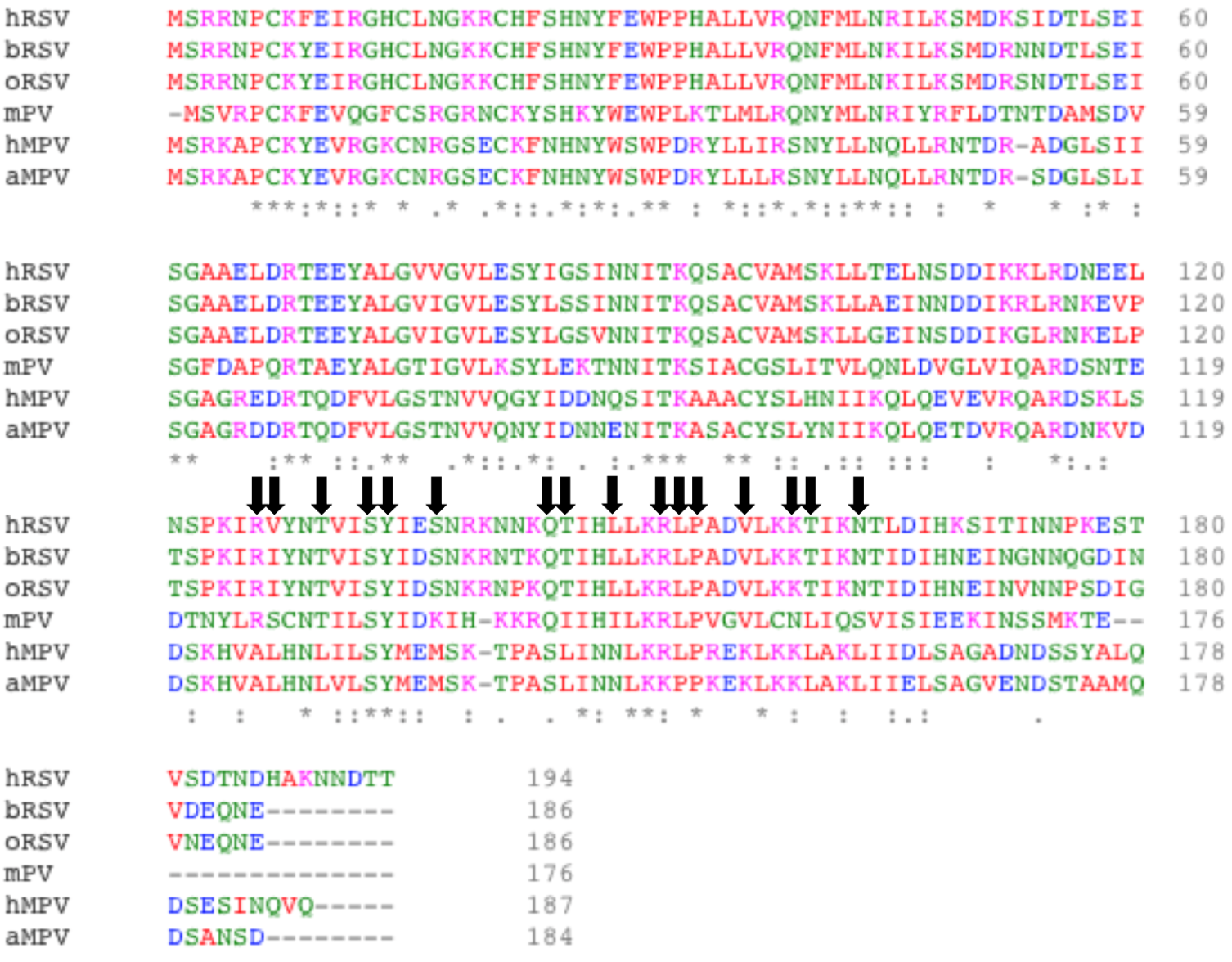

Supplement: FIG S2 [file mbo005184162sf2.tif]

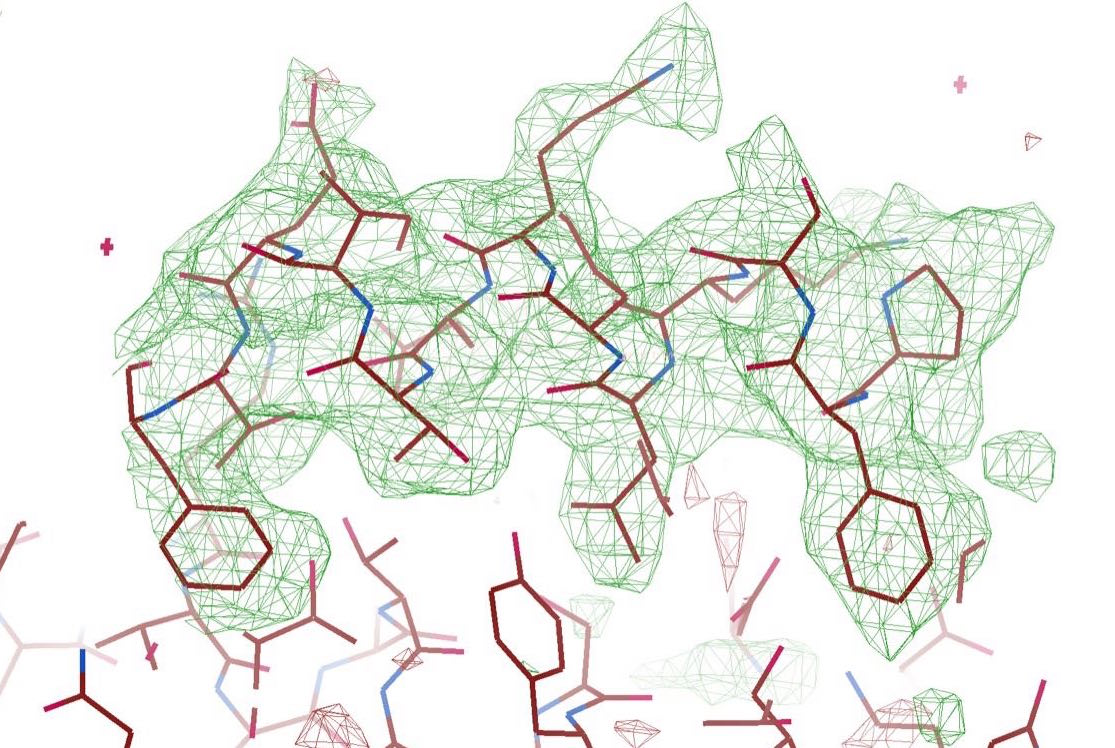

Supplement: FIG S3 [file mbo005184162sf3.tif]

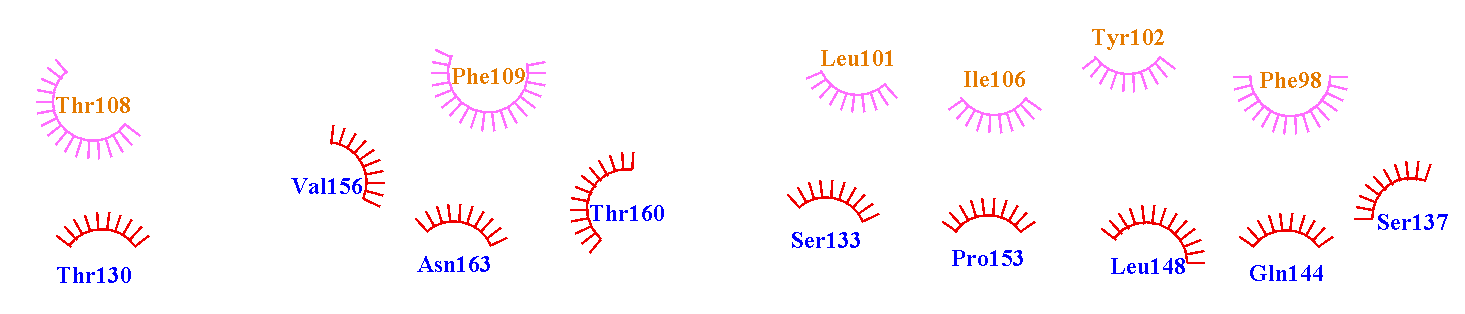

Supplement: FIG S4 [file mbo005184162sf4.tif]

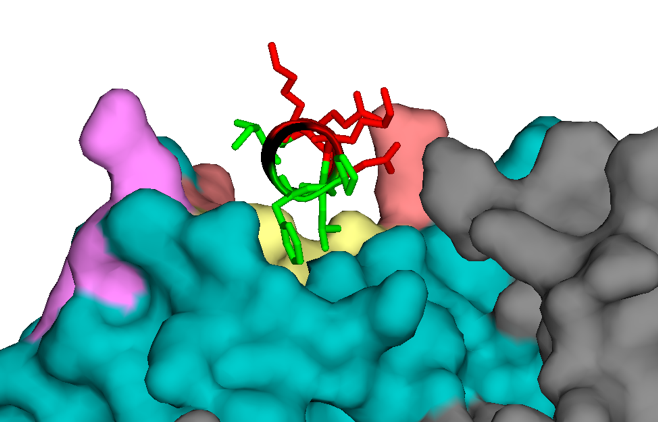

Supplement: FIG S5 [file mbo005184162sf5.tif]
